# Surgical Staging of Early Stage Endometrial Cancer: Comparison Between Laparotomy and Laparoscopy

**DOI:** 10.4021/wjon743w

**Published:** 2014-01-16

**Authors:** Murat Api, Semra Kayatas, Aysen Telce Boza, Hakan Nazik, Cevdet Adiguzel, Kadir Guzin, Mustafa Eroglu

**Affiliations:** a Department of Obstetrics and Gynecology, Zeynep Kamil Women and Children Diseases Training and Research Hospital, Istanbul 34668, Turkey; b Department of Obstetrics and Gynecology, Adana Numune Training and Research Hospital, Adana 01240, Turkey; c Department of Obstetrics and Gynecology, Goztepe Training and Research Hospital, Istanbul 34730, Turkey

**Keywords:** Endometrium cancer staging, Lymph node count, Laparoscopy

## Abstract

**Background:**

The aim of the present study was to compare the laparotomy (LT) and laparoscopy (LS) in patients who undergone surgical staging for early stage endometrium cancer.

**Methods:**

Retrospective data were collected and analyzed for amount of intraoperative bleeding, complication rates, total resected and laterality specific number of lymph nodes and duration of operation in patients operated with either LT or LS.

**Results:**

Seventy-nine stage I endometrium cancer patients were found to be eligible for the trial purposes: 58 (73.4%) treated by LT and 21 (26.6%) treated by LS. The number of lymph nodes was similar in LT (8.9 ± 5.3) and LS (9.2 ± 4.8) (P = 0.8). In LT group, there was no difference in the number of lymph nodes between the right and left sides (10 ± 5.8 and 8.7 ± 4.8 respectively, P = 0.19); in LS group, the number of lymph nodes resected from the right side was higher than the left side (9.8 ± 5 and 7 ± 3.5 respectively, P = 0.039). The amount of intraoperative bleeding and hospitalization period were significantly higher in LT group. Seventy-nine patients had a median follow-up of 30 months. The two groups were similar for disease-free survival (P = 0.46, log rank test).

**Conclusions:**

There was no significant difference between the two methods in terms of number of total resected lymph nodes. In early stage endometrial carcinoma, LS has provided adequate staging and similar survival rates with LT.

## Introduction

Cancer of endometrium is the most common gynecologic malignancy in developed countries [1]. Approximately 75% of affected women are diagnosed at an early stage, that is, when the cancer is clinically confined to the uterus [2]. Endometrioid type is the most common histologic subtype and tends to have a favorable prognosis.

Endometrial carcinomas are staged surgically according to the International Federation of Gynecology and Obstetrics (FIGO) classification system [3] and the American Joint Committee on Cancer [4]. The standard staging approach to endometrial carcinoma was done via laparotomy (LT) with total extrafascial hysterectomy, bilateral salpingooferectomy and pelvic and/or paraaortic lymph node dissection. Altough laparoscopic surgical staging was first reported in 1990, endoscopic approach was not used commonly in patients with endometrial cancer [5]. Laparoscopic surgical staging procedure was still under investigation for its efficancy and safety measures.

The aim of the present study was to compare the effectiveness of surgical staging of early stage endometrial cancer between conventional LT and laparoscopy (LS).

## Material and Method

The retrospective data were collected from a total of 79 clinically early stage endometrial cancer patients who were operated between 2010 and 2013 by surgeons with experiences in both oncology and LS. Twenty-one patients were staged via LS and 58 patients via LT. Cytology on the entry into the peritoneal cavity, extrafascial hysterectomy with bilateral salpingooferectomy and pelvic, if needed paraaortic, lymph node dissection were carried out for comprehensive surgical staging of endometrial cancer. Pelvic lymphadenectomy was performed to all patients while paraaortic lymph node dissection was performed if there were grade 3 disease, non-endometrioid histology and existence of invasion more than 50% of myometrium. Pelvic lymph nodes were to be removed from distal one half of the common iliac artery down to the circumflex vein, anterior to obturator vein and surrounding iliac arteries and vein. The paraaortic lymph nodes were collected from overlying the vena cava and abdominal aorta, up to the inferior mesenteric artery and down to the midpoint of the common iliac artery.

Disease stages were assessed according to the FIGO 2009 endometrial cancer staging system.

The demographic parameters were collected for the two groups as age, BMI and histologic type. Two groups were compared according to amount of intraoperative bleeding, hospitalization period, complication rates, total resected and laterality specific number of lymph nodes and duration of operation. Intraoperative bleeding was subjectively measured by the surgeons and recorded to the files. Postprocedure follow-ups of the patients’ data were also collected retrospectively for recurrence and survival. Time between the date of diagnosis and the date of recurrence was defined as disease free survival (DFS).

The study protocol was approved by the Institutional Review Board. All statistical analyses were performed with the Statistical Package for the Social Sciences version 15.0 (SPSS Inc., Chicago, IL, USA). Qualitative data are expressed in percentage (%) and quantitative data are expressed as the means ± standard deviation. Differences between the means in normally distrubuted variables were performed by using Student’s t test. Chi-square test was done on categoric variables. The survival data were analyzed by Kaplan-Meier method. A P value of < 0.05 was accepted as statistically significant.

## Results

**Figure 1:**
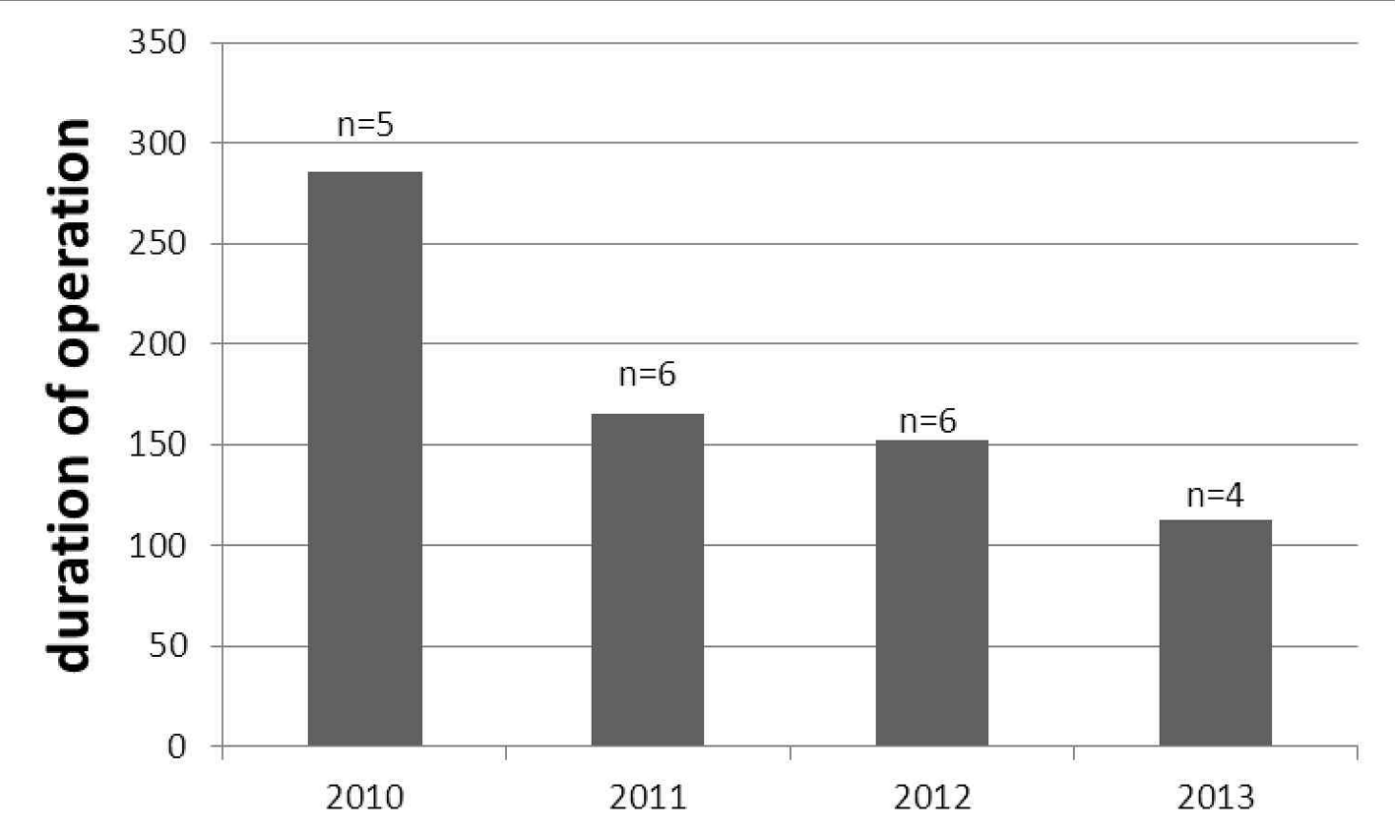
The decreasing duration of laparoscopic operation (minutes) among the years; number of operated patient shown with “n”.

The age, BMI, histopathologic type and intraoperative measures of the patients are shown in Table 1. The mean age of patients in both groups ranged 43 - 66 years. The histopathologic features were similar between groups. The mean durations of operations defined as first skin incision to skin closure were 180.4 ± 68.5 min for LS and 169.6 ± 46 min for LT (P = 0.4). The duration of operation was found to be decreased by years passed from 2010 to 2013 (Fig. 1). Estimated blood loss and hospitalization period were significantly higher in LT group (P < 0.0001 for both). The overall incidences of postoperative complications were similar between LS and LT (14.2% vs. 15%, respectively).

**Table 1:** The Demographic, Histopathologic Type and Intraoperative Measures of Patients

	Laparoscopy (n = 21)	Laparotomy (n = 58)	P value
Age (years), mean (range)	56 (46-66)	54 (43-65)	0.47
BMI, kg/m^2^, mean (± sd)	26.8 (± 2.1)	26.7 (± 2.6)	0.85
Tumor histology, n (%)			
Endometrioid	17 (80.9%)	53 (91.3%)	0.21
Others	4 (19.1%)	5 (8.7%)	
Tumor grade, n (%)			
Ι	12 (22.2%)	42 (77.8%)	0.21
ΙΙ	9 (39.1%)	14 (60.9%)	
ΙΙΙ	0	2 (100%)	
Duration of operation (min.), mean (range)	180.47 (112-248)	169.65 (123-215)	0.4
Intraoperative bleeding (mL), mean (± sd)	136.6 (± 33.9)	245.6 (± 68.7)	< 0.0001*
Postoperative complications			
Ileus	3	2	
Wound infection	0	4	
Evisceration	0	1	
Iliac artery injury	1	2	
Overall, n (%)	4 (14.2%)	9 (15%)	
Hospitalization period (day), mean (± sd)	5 (± 1.3)	8.5 (± 1.7)	< 0.0001*

BMI: body mass index; min.: minutes; sd: standard deviation. *P < 0.05.

All patients have undergone pelvic lympadenectomy, while paraaortic lymph nodes were documented from 28.5% of LS and 46.5% of LT group (Table 2). The average numbers of retrieved total pelvic lymph nodes were 9.2 (± 4.8) in LS and 8.9 (± 5.3) in LT (P = 0.8).

**Table 2:** Lymph Node Features of Operations

	Laparoscopy (n = 21)	Laparatomy (n = 58)	P value
Pelvic lymphadenectomy, n (%)	21 (100%)	58 (100%)	
Dissected pelvic lymph node count, mean (± sd)	9.2 (± 4.8)	8.9 (± 5.3)	0.8
Paraaortic lymphadenectomy, n (%)	6 (28.5%)	27 (46.5%)	
Dissected paraaortic lymph node count, mean (± sd)	3.1 (± 5.6)	4.1(± 6)	0.51

sd: standard deviation. P < 0.05.

**Figure 2:**
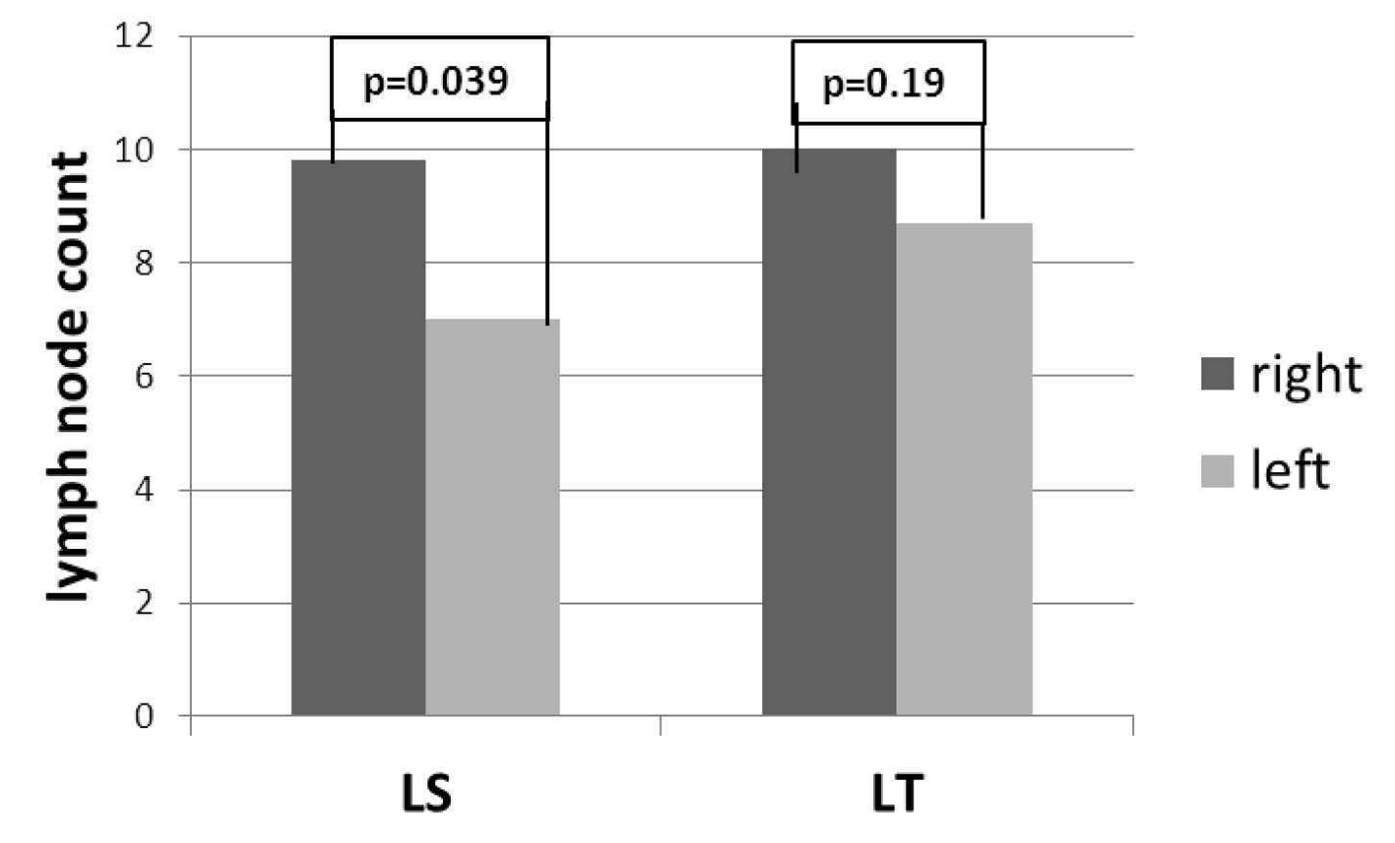
Laterality specific lymph node count between LS and LT.

In LT group, there was no difference in the number of lymph nodes between the right and left sides (10 ± 5.8 and 8.7 ± 4.8 respectively, P = 0.19); in LS group, the number of lymph nodes resected from the right side was higher than the left side (9.8 ± 5 and 7 ± 3.5 respectively, P = 0.039) (Fig. 2).

Two operations were started as LS and converted to LT due to poor exposure and excessive bleeding due to iliac artery injury; these were included to LT group.

**Figure 3:**
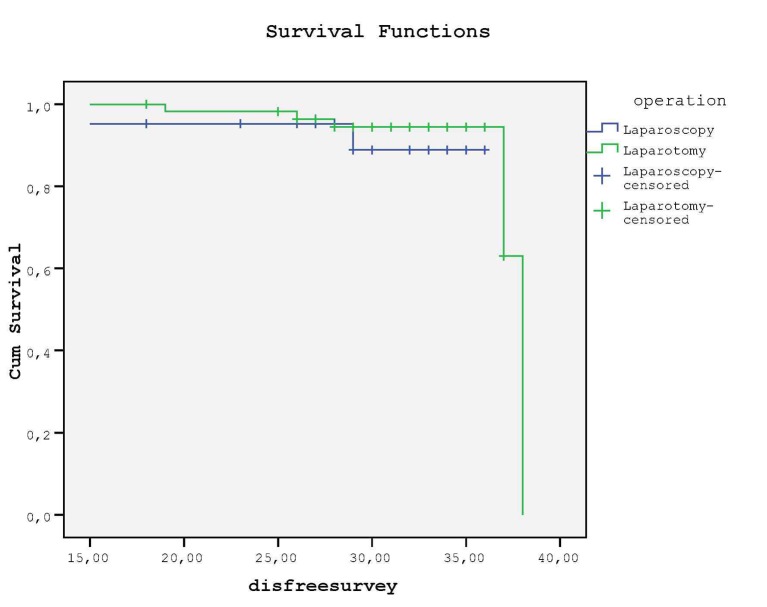
There is no statistical difference between groups for DFS.

Kaplan-Meier curve revealed that no difference was detected for DFS between groups (P = 0.46, log rank test) (Fig. 3); in LS group 2 (9.5%) and in LT group 5 (8.6%) relapses occured at the end of follow-up (mean 29.9 months vs. 30.1 months, respectively). None of the patients died due to recurrence or no death was reported during our clinical follow-up of 30 months.

## Discussion

The findings of the present study suggest that the staging of endometrial carcinoma via LS was as efficient and safe as LT because of similar complication rates, duration of operation, survival rates and sufficient staging.

According to the result of our study, duration of laparoscopic staging was no longer than LT and also we observed that duration of operation was decreased among the years suggesting with increasing experience of surgeons on LS. In the LAP2 [6] study, Walker et al found mean operation time of laparoscopic staging was significantly higher than LT (204 min vs. 130 min), while Taskin et al (2012), a prospective study from Turkey, reported that laparoscopic staging was no longer than LT and they achieved better dissection and exposure of pelvic spaces with increasing number of laparoscopic operations leading to shortened operation time [7].

In a randomized trial, Mourits et al [8] found similar complication rates between LS and LT (14.6% vs. 14.9%, respectively) while in LAP2 study [6], LT group encountered more adverse effect in postoperative period (21% in LT vs. 14% in LS). A meta-analysis of four randomized trials that compared surgical staging with laparoscopically assisted vaginal or total laparoscopic hysterectomy versus LT [9, 10] found that LS significantly took longer time (198 vs. 132 min), but resulted in fewer perioperative complications, decreased blood loss (267 mL less) and shorter hospital stays (3 vs. 4 days). In our study, postoperative complication rates were similar between groups (15% in LT vs. 14.2% in LS), but LS was found to be associated with decreased intraoperative blood loss and hospitalization stay.

In the literature, conversion to LT due to intraoperative complications was reported between 1.4% and 23.7% [6, 11]. In our study, two of 23 (8.6%) cases were converted to LT.

In a recent review about survival rate after 3-year follow-up revealed that DFS was similar between LS and LT groups (80-91% after 36 - 78 months of median follow-up vs. 81-92% after 30 - 80 months of median follow-up, respectively) [12]. Also Malur et al (2001), Obermair et al (2004) and Seracchioli et al (2005) found that survival rate was similar between groups [11, 13, 14]. In our study, only DFS was analyzed due to the absence of death in a follow-up period of 30 months, and we found that staging procedure had no effect on DFS.

In our study, the number of removed lymph nodes from pelvic and paraaortic areas was found to be similar between groups (P = 0.8 vs. P = 0.16, respectively). Both the LAP2 study [6] and the meta-analysis of Palombo et al [9] reported similar number of pelvic and paraaortic lymph nodes in both laparoscopic and laparotomic staging.

Lutman et al (2006) suggested to retrieve ≥ 12 pelvic lymph nodes in patients with FIGO stage 1 and 2 endometrial cancer who have high risk histology because of better survival rate [15]. In our study, 11.3% of the patients were with high risk histology and mean pelvic lymph node count of this group was 21.3 as recommended in the literature.

Although our experience was limited to the 79 early stage endometrial cancer patients, the right side prevelance of lymph nodes was obvious either by LT or LS. Since the data collected retrospectively, there was no effort in the collection of right and left side pelvic lymph nodes according to a standardized fashion. There might be a chance in these observations; however, one explanation to the right side dominance of pelvic lymph nodes was the position and anatomic location of sigmoid colon on the left side. The surgeon might have started to collect lymph nodes over the common iliac arteries below the level of sigmoid mesenterium on the left side but surgeon could perform easier dissection over the right common iliac artery. So the right side predominance could be based on technical reason. Ghezzi et al (2005) reported that the difference in lymph node distribution in favor of the right side was invariably observed in all subgroups, irrespective of indication to surgery (endometrium, ovarian or cervical cancer), nodal group, type of surgical procedure (LT or LS) and patient’s BMI [12]. In our study, the right side predominance was found to be statistically significant in LS group. Although Ghezzi et al thought that the lymph node asymmetry unlikely depends on surgical factors, our experience suggested that it seems to be related to better surgical exposure with both the advantage of LS and absence of sigmoid colon on right side.

Limitations of our findings were the number of subjects in each group and the retrospective nature of our study. There was a need for further prospective randomized trial with sufficient amount of participants still remained.

In conclusion, there was no significant difference between the LT and LS in terms of number of total resected lymph nodes. In early stage endometrial carcinoma, LS has provided adequate staging and similar survival rates with LT. In future, LS may become mainly preferred procedure in the endometrial cancer staging.
